# Successful Pharmacological Management of a Severe Suicide Attempt

**DOI:** 10.1192/j.eurpsy.2025.1346

**Published:** 2025-08-26

**Authors:** O. Martin Santiago, B. Arribas-Simon, O. Segurado-Martin, M. Rios-Vaquero, M. Calvo-Valcarcel, M.-P. Pando-Fernandez

**Affiliations:** 1 Hospital Clinico Universitario de Valladolid, Valladolid, Spain

## Abstract

**Introduction:**

Psychotic depression, a severe subtype of major depressive disorder with delusions or hallucinations, increases suicide risk due to distressing symptoms and hopelessness. Suicide attempts in psychotic depression can be severe and violent. Combining antidepressants and antipsychotics shows promise in reducing suicidal ideation and improving prognosis. This case presents a patient with a severe suicide attempt and self-harm in the context of psychotic depression, highlighting successful treatment with a combination of antidepressants and antipsychotics.

**Objectives:**

To present a case study of a patient with a depressive episode that progressed to psychotic features.

**Methods:**

A comprehensive literature search was conducted to identify relevant studies on the treatment of depression with psychotic features. A case report was then developed, detailing the patient’s clinical presentation, diagnosis, and treatment regimen.

**Results:**

A 53-year-old male was hospitalized following a serious suicide attempt. The patient had a history of a recent work-related accident, leading to a depressive episode that progressed to psychotic features, including delusions of guilt and economic ruin, attempted suicide using a firearm, leading to significant self-inflicted injuries. Emergency surgical intervention was required for tendon and arterial damage. Psychiatrically, the patient exhibited profound hopelessness, delusional guilt, and active suicidal ideation. Following hospital admission, the patient was treated with a combination of sertraline, olanzapine, and mirtazapine, which resulted in significant improvement in mood, a reduction of delusions, and cessation of suicidal ideation over a three-weeks period. The patient returned to social activities and expressed interest in resuming his professional responsibilities, with no recurrence of psychotic symptoms or suicide attempts.

**Conclusions:**

This case illustrates the severity of suicidal behavior in psychotic depression and the critical importance of combining antidepressants with antipsychotics for effective management. Research has consistently shown that psychotic depression carries a heightened risk of severe suicide attempts due to the intensity of delusions and hopelessness. Antidepressant-antipsychotic combinations, particularly those involving selective serotonin reuptake inhibitors (SSRIs) like sertraline, and atypical antipsychotics such as olanzapine, have demonstrated efficacy in reducing both depressive and psychotic symptoms, thereby mitigating suicide risk. In this case, the patient’s marked improvement and remission of psychotic features underscore the role of combined pharmacotherapy in stabilizing mood and preventing future suicidal behavior.

**Disclosure of Interest:**

None Declared

